# Functional cure of a young child with chronic hepatitis B cirrhosis treated by pegylated interferon α combination therapy: A case report

**DOI:** 10.1097/MD.0000000000041103

**Published:** 2025-01-10

**Authors:** Yu Gan, Hongfei Zhang

**Affiliations:** a Pediatric Hepatology, Hepatobiliary Pancreatic Center, Beijing Tsinghua Changgung Hospital, Tsinghua University, Beijing, China; b Jumei Doctor Group Medical (Shenzhen) Co., Ltd, Shenzhen, China.

**Keywords:** child, chronic hepatitis B, cirrhosis, functional cure, nucleos(t)ide analogues, pegylated interferon α

## Abstract

**Rationale:**

Current research on antiviral treatment in children is relatively limited, especially in children under 1 year old.

**Patient concerns:**

Liu XX, an 8-month-old infant (case number: 3001120473), presented to the hospital in August 2016 with a chief complaint of being “hepatitis B surface antigen positive for 8 months and experiencing abnormal liver function for 5 months.”

**Diagnoses:**

The patient was diagnosed as chronic hepatitis B cirrhosis (G3S3-4) with active compensatory phase.

**Interventions:**

The treatment regimen commenced with lamivudine (LAM) for the initial 8 weeks, followed by the addition of interferon α (IFNα) after 1 year of age. At 2 years old, LAM was substituted with entecavir, and at 3 years old, IFNα was replaced with pegylated interferon α (PEG IFNα).

**Outcomes:**

After 8 weeks of LAM monotherapy, Liu XX experienced hepatitis B e antigen loss. Subsequently, after 36 weeks of IFNα add-on therapy, hepatitis B virus DNA became undetectable, and after 48 weeks of switching to PEG IFNα treatment, hepatitis B surface antigen loss was observed. Remarkably, following 50 weeks of drug discontinuation, the child remained functionally cured.

**Lessons:**

Chronic hepatitis B virus-infected infants and young children can achieve durable functional cure with PEG IFNα-based individualized therapy. This case provides a valuable reference for the diagnosis and treatment of such patients.

## 1. Introduction

Chronic hepatitis B virus (HBV) infection poses a significant health burden, often leading to liver inflammation and potentially progressing to cirrhosis or hepatocellular carcinoma. Extensive clinical trials on chronic hepatitis B (CHB) treatment in adult patients with low viral load and hepatitis B surface antigen (HBsAg) levels have demonstrated HBsAg loss rates ranging from 30% to 80% with the use of pegylated interferon α (PEG IFNα) therapy.^[1,2]^ In contrast, the management of HBV-infected children presents distinct challenges, as they are more susceptible to developing CHB, which greatly impacts their overall health and growth, garnering widespread attention. The clinical management of children with CHB involves complexities related to determining the appropriate treatment initiation time, selecting suitable drugs, establishing optimal therapeutic dosages and durations, and minimizing adverse events, among other considerations. Moreover, available drugs and guideline recommendations specifically tailored to children remain relatively limited. Recent evidence has suggested that pediatric patients treated with (PEG) IFNα-dominant therapy achieve HBsAg loss rates ranging from 20% to 50%, and initiating antiviral therapy early in the disease course predicts greater treatment benefits.^[3,4]^ Notably, a prospective real-world study demonstrated that 14 pediatric patients with S3/4 fibrosis exhibited significant improvement in liver histology with lamivudine (LAM) combined with IFNα therapy, irrespective of HBsAg loss.^[5]^ However, data for patients under 1 year of age are limited. In this study, we share our experience with achieving functional cure in an 8-month-old patient suffering from chronic hepatitis B cirrhosis. We hope that this valuable insight will provide useful references for the clinical management of such young patients.

## 2. Case presentation

**Figure 1.**
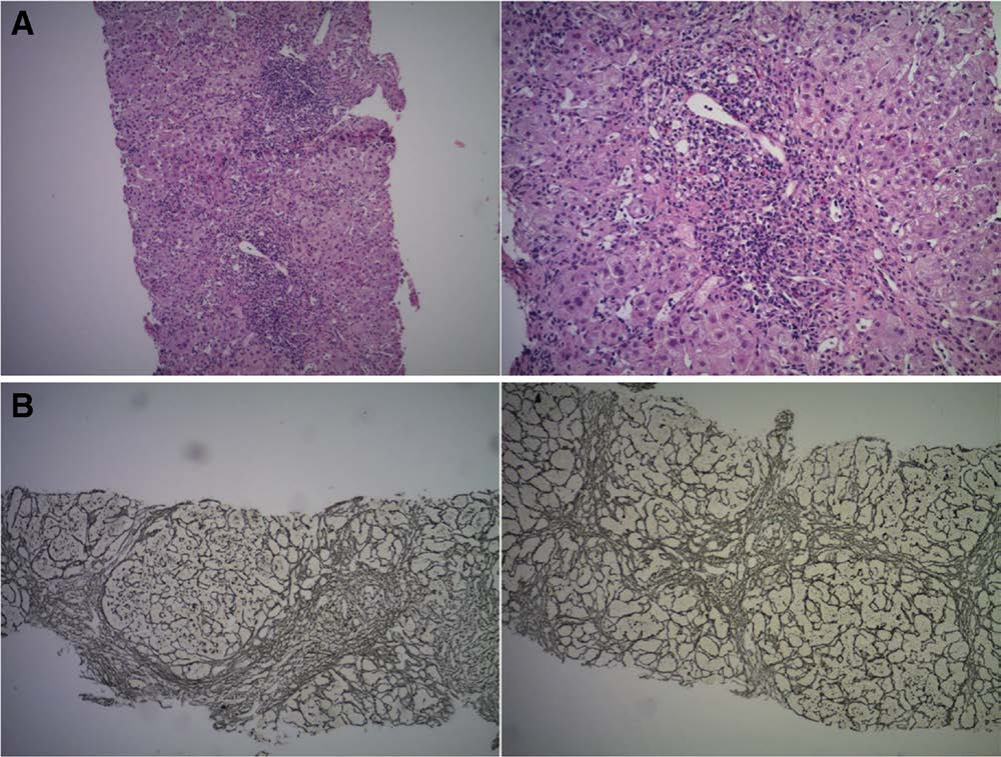
Liver histology of the patient. (A) Hematoxylin-eosin staining. (B) Reticular fibrous staining.

In August 2016, an 8-month-old female child presented to our hospital with a chief complaint of being “HBsAg positive for 8 months and experiencing abnormal liver function for 5 months.” The patient had a noteworthy family history of CHB, with her mother, uncle, maternal grandfather, and 2 brothers of her maternal grandfather all afflicted by CHB. Tragically, her maternal grandfather had succumbed to hepatocellular carcinoma. Her mother was a patient with CHB but did not receive antiviral treatment. Following birth, the patient had received routine hepatitis B vaccination and hepatitis B immunoglobulin. Upon physical examination, the child exhibited moderate nutrition, normal development, and no discernible positive signs. The results of outpatient examinations were as follows: HBV DNA 1.65 × 10^7^ IU/mL, HBV genotype B; HBsAg 5446.00 IU/mL, hepatitis B surface antibody (HBsAb) < 2 IU/L, hepatitis B e antigen (HBeAg) 1.18 COI, hepatitis B e antibody 0.069 COI, hepatitis B core antibody 0.006 COI. Furthermore, liver function tests revealed alanine aminotransferase (ALT) at 41 U/L, aspartate aminotransferase at 151 U/L, cholinesterase at 4104 U/L, and alpha-fetoprotein > 1210 ng/mL. Abdominal ultrasonography exhibited no significant abnormalities in the liver, gallbladder, pancreas, or spleen, while coagulation, renal, and thyroid functions were all within the normal range. The liver biopsy revealed disordered hepatic lobule structure, with the presence of local pseudolobule formation. Diffuse hydropic and balloon-like degeneration of hepatocytes were observed, along with scattered spotty necrosis, fusion focal necrosis, and bridging necrosis. Additionally, there was significant inflammation in the hepatic sinusoids, accompanied by Kupffer cells. Enlargement of the portal area with fibrous tissue hyperplasia and fibrous septum formation was also evident. The biopsy displayed infiltration of numerous mixed inflammatory cells, including eosinophils, and some portal areas exhibited lymphocyte infiltration. Lymphoid follicular structures and moderate interfacial inflammation were also noted (Fig. 1). Based on these findings, the patient was diagnosed with chronic hepatitis B cirrhosis in the active compensatory phase (G3S3-4).

**Figure 2.**
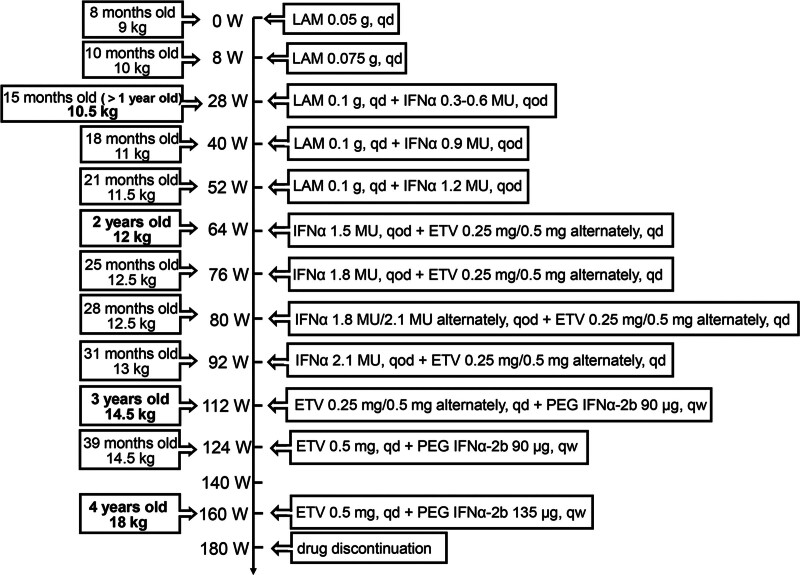
Treatment process. W = weeks; qd = quaque die; qod = quaque omni die; qw = quaque week; LAM = lamivudine; ETV = entecavir; IFNα = interferon α; PEG IFNα = pegylated interferon α.

**Figure 3.**
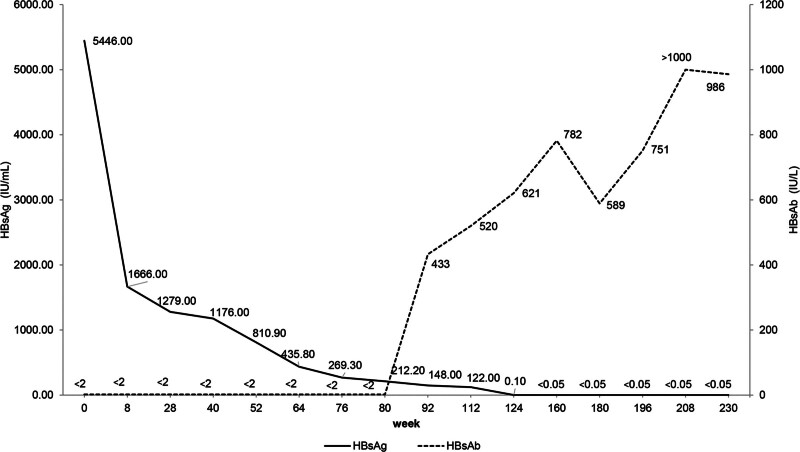
Changes of HBsAg and HBsAb during treatment and follow-up. HBsAg = hepatitis B surface antigen; HBsAb = hepatitis B surface antibody.

Since the patient’s CHB had progressed to early cirrhosis, prompt initiation of antiviral therapy was crucial to control the disease. After thorough communication with the family and obtaining informed consent, antiviral therapy was initiated in August 2016. As per the Chinese guideline of prevention and treatment for chronic hepatitis B (2015 update), antiviral drugs approved for pediatric patients include nucleos(t)ide analogues and IFNα; however, IFNα is not recommended for children under 1 year of age.^[6]^ Given the patient’s age of 8 months, an initial oral dose of LAM at 0.05 g/day was administered. After 8 weeks of treatment, significant progress was observed, with HBV DNA decreasing to 356 IU/mL, HBsAg reducing to 1666.00 IU/mL, and HBeAg seroconversion being achieved. Subsequently, the LAM dose was adjusted to 0.075 g/day based on the patient’s weight and age. LAM monotherapy continued to 28 weeks, resulting in a further decline of HBV DNA to 80 IU/mL, although HBsAg showed only a slight reduction. Upon reaching 1 year of age, IFNα was added to the treatment regimen at a dosage of 0.3 to 0.6 MU subcutaneously once every other day, with the dose gradually increasing with age and weight. At 64 weeks (36 weeks of IFNα add-on therapy), a complete virological response was achieved, with HBV DNA measuring <20 IU/mL and HBsAg declining to 435.80 IU/mL. At this point, when the patient turned 2 years old, LAM was switched to entecavir administered at 0.25 mg/0.5 mg alternately daily, while IFNα treatment continued at an escalated dose. At 112 weeks of treatment, HBsAg decreased to 122.00 IU/mL, and a high level of HBsAb was detected. Simultaneously, the patient reached 3 years of age, prompting a switch from IFNα to PEG IFNα-2b (Xiamen Amoytop Biotech Co., Ltd, Xiamen, China) at 90 μg/week, in line with the approvals by the U.S. Food and Drug Administration and European Medicines Agency (EMA) for PEG IFNα use in children aged 3 years and older in 2017.^[7,8]^ At 160 weeks (48 weeks of PEG IFNα combination therapy), HBsAg loss and seroconversion were achieved, with a sustained increase in HBsAb to 782 IU/L, leading to functional cure. Thereafter, PEG IFNα-2b at 135 μg/week, combined with entecavir at 0.5 mg/day, was continued for an additional 20 weeks, during which HBsAg, HBeAg, and HBV DNA remained persistently negative. Eventually, all antiviral drugs were discontinued. During the follow-up period of 50 weeks, the functional cure was maintained, and HBsAb levels consistently elevated above 900 IU/L. The specific treatment regimen and monitoring of various indicators are depicted in Figures 2 and 3, and Table 1.

**Table 1:** Changes of monitoring indicators during treatment and follow-up.

Treatment timepoint (W, weeks)	HBsAg (IU/mL)	HBsAb (IU/L)	HBeAg (COI)	HBeAb (COI)	HBV DNA (IU/mL)	ALT (U/L)	WBC (×10^9^/L)	ANC (×10^9^/L)	PLT (×10^9^/L)
0 W	5446.00	<2	1.18	0.026	1.39 × 10^5^	38	10.84	2.000	442
8 W	1666.00	<2	**0.15**	0.031	356	19	10.13	1.710	322
28 W	1279.00	<2	0.11	0.006	80	23	3.69	1.700	296
40 W	1176.00	<2	0.10	0.003	<20	20	3.38	1.800	307
52 W	810.90	<2	0.10	0.002	49	47	11.39	1.900	431
64 W	435.80	<2	0.12	0.003	**<20**	19	3.21	1.300	271
76 W	269.30	<2	0.11	0.005	<20	18	/	/	/
80 W	212.20	<2	0.12	0.003	<20	16	3.27	1.700	282
92 W	148.00	**433**	0.10	0.002	<20	31	5.59	1.900	236
112 W	122.00	520	0.10	0.003	<20	52	5.64	1.800	239
124 W	0.10	621	0.11	0.003	<20	32	/	/	/
160 W	**< 0.05**	782	0.10	0.003	<20	12	/	/	/
180 W	< 0.05	589	-	**+**	<500	/	/	/	/
196 W	< 0.05	751	-	+	<500	/	/	/	/
208 W	< 0.05	>1000	-	+	<500	/	/	/	/
230 W	< 0.05	986	-	+	<500	17	/	/	/

ALT = alanine aminotransferase; ANC = absolute neutrophil count; HBeAb = hepatitis B e antibody (> 1 COI, negative); HBeAg = hepatitis B e antigen (<1 COI, negative); HBsAb = hepatitis B surface antibody (< 10 IU/L, negative); HBsAg = hepatitis B surface antigen (< 0.05 IU/mL, negative); HBV DNA = hepatitis B virus DNA (<20 IU/mL, negative) ; / = not detected; PLT = platelet; WBC = white blood cell.

Remarkably, throughout the treatment course, the patient did not experience any noticeable adverse reactions such as fever, loss of appetite, or growth retardation. To prevent neutropenia during IFNα therapy, IFNα was initially administered in combination with leucogen tablets at a dose of 20 mg twice daily. On rechecking the absolute neutrophil count after 10 days, it was found to be 0.76 × 10^9^/L, leading to an adjustment in the dosage of leucogen to 40 mg daily. With this prophylactic adjustment, neutropenia was maintained within a tolerable range, and IFNα dosage did not require reduction. Furthermore, platelet count, thyroid function, and autoantibodies showed no abnormalities during treatment, indicating good tolerance to the therapy.

## 3. Discussion

In China, mother-to-child transmission accounts for 30% to 50% of HBV infections. The implementation and widespread use of the hepatitis B vaccine in the national immunization program since the 1990s have substantially reduced the risk of HBV infection in children. However, there is still a considerable number of HBV-infected children, with an estimated 1.8 million children under the age of 15 being infected nationwide and approximately 50,000 newborns acquiring HBV each year.^[9]^ Childhood HBV infection remains a significant public health concern.

It is widely believed that early HBV infection leads to greater immune tolerance, resulting in many pediatric HBV-infected patients going undiagnosed and untreated due to vague clinical symptoms. Nonetheless, a considerable proportion of pediatric patients are susceptible to breaking through this immune tolerance, leading to varying degrees of disease progression. Without treatment, approximately 90% of perinatally infected patients develop CHB, and 30% to 40% of young children progress to CHB, compared to only 10% of adults.^[10]^ Various studies have demonstrated that over 60% of pediatric CHB patients, including those diagnosed in the immune-tolerant phase, exhibit inflammation grades higher than G2, over 30% have fibrosis stages exceeding S2, and approximately 10% have cirrhosis based on liver biopsy results.^[11–14]^ In this case, an 8-month-old patient with CHB presented with early cirrhosis, indicating significant disease progression even in infants. This highlights the importance of vigilance among healthcare professionals and patients, as disease severity can manifest early in life and necessitate timely intervention.

The spontaneous HBsAg loss rate in children with CHB is exceedingly low, ranging from 0.6% to 1% per year.^[15]^ Therefore, prompt initiation of treatment is crucial to improve the prognosis, prevent disease progression, and reduce the number of CHB adults arising from chronic HBV-infected children. According to the Chinese guidelines for the prevention and treatment of chronic hepatitis B (version 2022), children with advanced liver disease or cirrhosis, irrespective of age, should receive timely antiviral therapy. Additionally, children with positive HBV DNA and ALT levels below the upper limit of normal (immune tolerance phase) should also be considered for antiviral therapy, particularly those aged 1 to 7 years who are categorized under the “so-called immune tolerance phase.”^[16]^ Several existing studies have shown promising results regarding HBsAg loss rates in pediatric CHB patients treated with (PEG) IFNα-based therapy. For instance, Poddar et al administered IFNα combined with LAM for 6 months in immune-tolerant children with chronic HBV infection, averaging 6 years of age, and achieved HBsAg loss in 21.4% of cases.^[17]^ Additionally, Fan and Liu et al observed that approximately 50% of infected children over 2 years of age attained HBsAg loss after 48 or 52 weeks of PEG IFNα monotherapy.^[18,19]^ Moreover, several subsequent studies reported HBsAg loss rates ranging from 35% to 65% in children aged 1 to 18 years treated with IFNα monotherapy or in combination/sequence with LAM.^[5,12,20]^ Evidently, pediatric CHB patients undergoing (PEG) IFNα-based therapy have a relatively high likelihood of achieving HBsAg loss. Moreover, a meta-analysis revealed that children aged ≤ 6 years had significantly higher HBeAg and HBsAg seroconversion rates compared to those > 6 years (60% vs 37%, *P* = .20; 65% vs 42%, *P* < .05) after PEG IFNα dominant therapy, indicating a superior clinical cure rate in children treated at an earlier age.^[4]^ Furthermore, a real-world study assessed the impact of initiation time on treatment outcomes in HBV-infected infants, demonstrating a 12-month HBsAg loss rate of 83% and 36% in patients initiated on antiviral therapy before and after 1 year of age, respectively.^[21]^ In conclusion, antiviral therapy offers significant benefits for children with CHB, but current evidence remains insufficient, particularly for infants and young children. There is also a lack of standardized protocols regarding treatment strategies, courses, and the duration of consolidation therapy, necessitating further extensive research to validate and supplement the available evidence.

The achievement of a functional cure in this 8-month-old child through individualized antiviral therapy serves as strong evidence of cure in infants and young children. Given the patient’s initial presentation with abnormal liver function and early cirrhosis, (PEG) IFNα dominant antiviral therapy was promptly administered, with drug type and dose adjustments made based on the child’s age and weight. The outcome demonstrated a complete virological response after 36 weeks of LAM combined with IFNα therapy, followed by HBsAg loss achieved after 48 weeks of transitioning to PEG IFNα. Furthermore, the patient maintained functional cure throughout the 50-week follow-up period after 20 weeks of consolidation treatment. This experience underscores the significance of tailoring drug types and doses according to the age and weight of pediatric patients, as they are in a crucial phase of growth and development. Individualized treatment with appropriate dosages and courses appears to lead to better outcomes in pediatric patients.

Safety and drug resistance concerns associated with long-term treatment in pediatric CHB patients have been raised. It is often believed that pediatric patients, due to their young age, may be more susceptible to adverse reactions and drug resistance. However, existing studies have found that the incidence of adverse events such as elevated ALT and neutropenia in children treated with PEG IFNα is similar to that in adults.^[19,22]^ In this case, the patient was closely monitored with appropriate prevention measures in place, and no significant abnormalities in physiological indicators and examinations were observed throughout the study. The incidence and severity of adverse events were lower than that seen in adults, indicating that long-term treatment in pediatric patients can be deemed safe and can be further improved with appropriate preventive measures.

As functional cure in chronic HBV-infected adults rapidly develops and advances, the focus is also turning towards the special group of children with CHB. This case serves as a valuable reference for the diagnosis and treatment of infants and young children. With ongoing progress and further exploration, more high-quality medical evidence will undoubtedly emerge to guide and standardize the clinical management of children with CHB.

## Author contributions

**Data curation:** Yu Gan.

**Investigation:** Yu Gan, Hongfei Zhang.

**Methodology:** Yu Gan, Hongfei Zhang.

**Project administration:** Yu Gan, Hongfei Zhang.

**Writing – original draft:** Yu Gan.

**Writing – review & editing:** Hongfei Zhang.
